# Linear Tapered Slot Antenna for Ultra-Wideband Radar Sensor: Design Consideration and Recommendation

**DOI:** 10.3390/s19051212

**Published:** 2019-03-09

**Authors:** Vincent Tseng, Cheng-Yuan Chang

**Affiliations:** Department of Electrical Engineering, Chung Yuan Christian University, Jhongli, Taoyuan 320, Taiwan; vince747@gmail.com

**Keywords:** ultra-wideband (UWB), tapered slot antenna (TSA), linear tapered slot antenna (LTSA), radar, sensor

## Abstract

Radar is a type of wireless, noncontact sensor that does not need to be placed on or near a test object for detection. A key component of any radar sensor is the antenna. Among different types of antennas, the linear tapered slot antenna (LTSA) is a wideband antenna that has the advantages of small size, design simplicity, and easy adaptation to an array. This study examined and analyzed the 10 primary parameters that define the LTSA design when operated in the ultra-wideband (UWB) frequency range. The study method involved varying each of the 10 parameters to discern how the variations impact the three critical characteristics of an antenna, namely, (1) return loss, (2) the far field radiation pattern on the E-plane, and (3) the far field radiation pattern on the H-plane. By analyzing the changes in these critical characteristics, a set of design recommendations for the 10 parameters was developed for the LTSA.

## 1. Introduction

A radar sensor is a noncontact sensor that can see through walls, which makes it perfect for various applications, such as fall detection for the elderly [1], 3D tracking [2], etc. Among the frequencies on which a radar sensor can be operated, ultra-wideband (UWB) is a good choice due to its wide spectrum and well-defined specification [3] for implementation. A key component of any radar sensor is its antenna. For UWB directional radar, previous studies have commonly deployed four types of antenna: microstrip disc [4,5,6,7], tapered slot [8], sinuous [9], and Yagi–Uda [10]. The microstrip disc antenna generates two main lobes [5], not just one, which makes it a bidirectional antenna instead of unidirectional, like the tapered slot, sinuous, or Yagi–Uda antennas. Several designs have been devised to turn the bidirectional nature of the microstrip disc antenna into being unidirectional [4,6,7] by strategically placing a ground plate to absorb the undesirable main lobe. These designs successfully eliminated the undesirable main lobe; however, side effects were introduced along the way, such as that the remaining main lobe’s direction became frequency dependent. On the other hand, the sinuous antenna design [9] is very complex when compared with the tapered slot antenna (TSA), involving multiple curvatures and angles. The Yagi–Uda [10] antenna design, although less complex than the sinuous (i.e., only needing to consider the shape of the segments and the distance between segments), still requires multiple segments and is more complicated than the tapered slot antenna.

In summary, the TSA has the following advantages over the other three antenna designs:

1. Being unidirectional,
2. Involving only one segment—slot,
3. Having no curvatures or angles, or only simple ones,
4. Able to change half-power beamwidth (HPBW) by changing the slot length, unlike other antennas which require a complete redesign, and
5. Integrating easily with a monolithic microwave integrated circuit (MMIC), making it well suited for antenna array implementation [11].

Due to the aforementioned advantages, this study focused on the TSA. In this group, three types of TSA are routinely designed: Vivaldi, linear tapered (LTSA), and constant wide (CWSA). All three share 10 primary parameters in design. The LTSA requires no additional parameters beyond the primary, while Vivaldi and CWSA do, such as the curve’s amplitude and the magnification factor for Vivaldi, and the feed taper length, constant length, end taper length, constant width, etc., for CWSA. The additional parameters provide finer controls on HPBW and side lobe level but come at the cost of antenna gain. For example, the baseline model of this study (shown below) has a gain of 10.68 dB, which is better than the antipodal Vivaldi antenna designed by Osman et al. [12] (gain of 4.3 dB) or Moosazadeh and colleagues [13,14] (gain of 8.5 dB). It is also better than the Vivaldi antenna designed by Zhang et al. [15] (gain of 6.7 dB) or Kerati et al. [16] (gain of 8 dB).

In this paper, the focus is on the 10 primary parameters that define the LTSA and are shared by Vivaldi and CWSA. Unlike previous studies which only focused on a certain set of these 10 parameters [8,11,17,18], this paper provides comprehensive recommendations for all 10 parameters, which, if followed, should result in a functional LTSA design and serve as a basis for further optimization, if desired.

This paper is organized as follows: Section 2 presents the 10 primary parameters and their assessment criteria in this study; Section 3 describes parameter variations and analyzes outcomes; Section 4 discusses results and recommends the operating range for each parameter; and Section 5 presents the conclusions.

## 2. Methods

**Figure 1:**
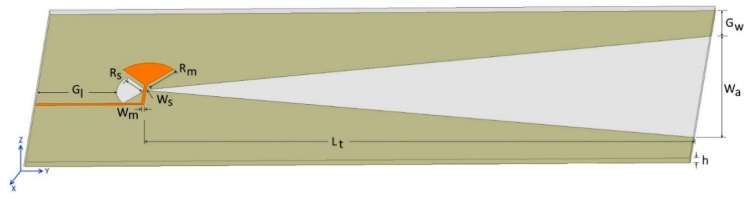
Parameters for a tapered slot antenna (TSA) on a double-sided printed circuit board (PCB) with a microstrip (orange color) on the top side and ground plane (olive color) on the bottom side.

A TSA, as shown in Figure 1, is defined by the following 10 primary parameters:

1. Slot width ($W_{s}$),
2. Microstrip width ($W_{m}$),
3. Slot stub radius ($R_{s}$),
4. Microstrip stub radius ($R_{m}$),
5. Slot opening width ($W_{a}$),
6. Tapered length ($L_{t}$),
7. Ground width ($G_{w}$),
8. Ground length ($G_{l}$),
9. Substrate thickness (h), and
10. Substrate material.

These 10 primary parameters are shared by all TSAs. In this study, a baseline model consisting of the 10 primary parameters was created as a starting point to measure how variations of parameters impact antenna characteristics. Guided by findings from previous studies [8,11,17,18], the initial values of the parameters in this baseline model were derived (Table 1). Following this, values of the 10 parameters were varied individually to gauge their impact on return loss (S11), the far field radiation pattern on the E-plane (Figure 1, YZ plane), and the far field radiation pattern on the H-plane (Figure 1, XY plane), which are the three key antenna characteristics. 

### 2.1. Baseline Model

Although a range of values for these 10 parameters could be generated from previous studies, to observe the changes caused by varying the parameters, a set value needed to be assigned as a starting point for each parameter. The initial values in this baseline model were generated by restricting the known values of the 10 parameters with the following two conditions: (1) to have S11 ≤ −10 dB (10% power loss) over 90% of the UWB range, while the overshoot over −10 dB was selected to be ≤−8.5 dB (14.13% power loss), and (2) the center frequency of the baseline model was set at 6.85 GHz, the middle frequency point of the UWB range from 3.1 to 10.6 GHz. These two conditions set a reasonable boundary. Table 1 shows the settings of the 10 parameters.

**Figure 2:**
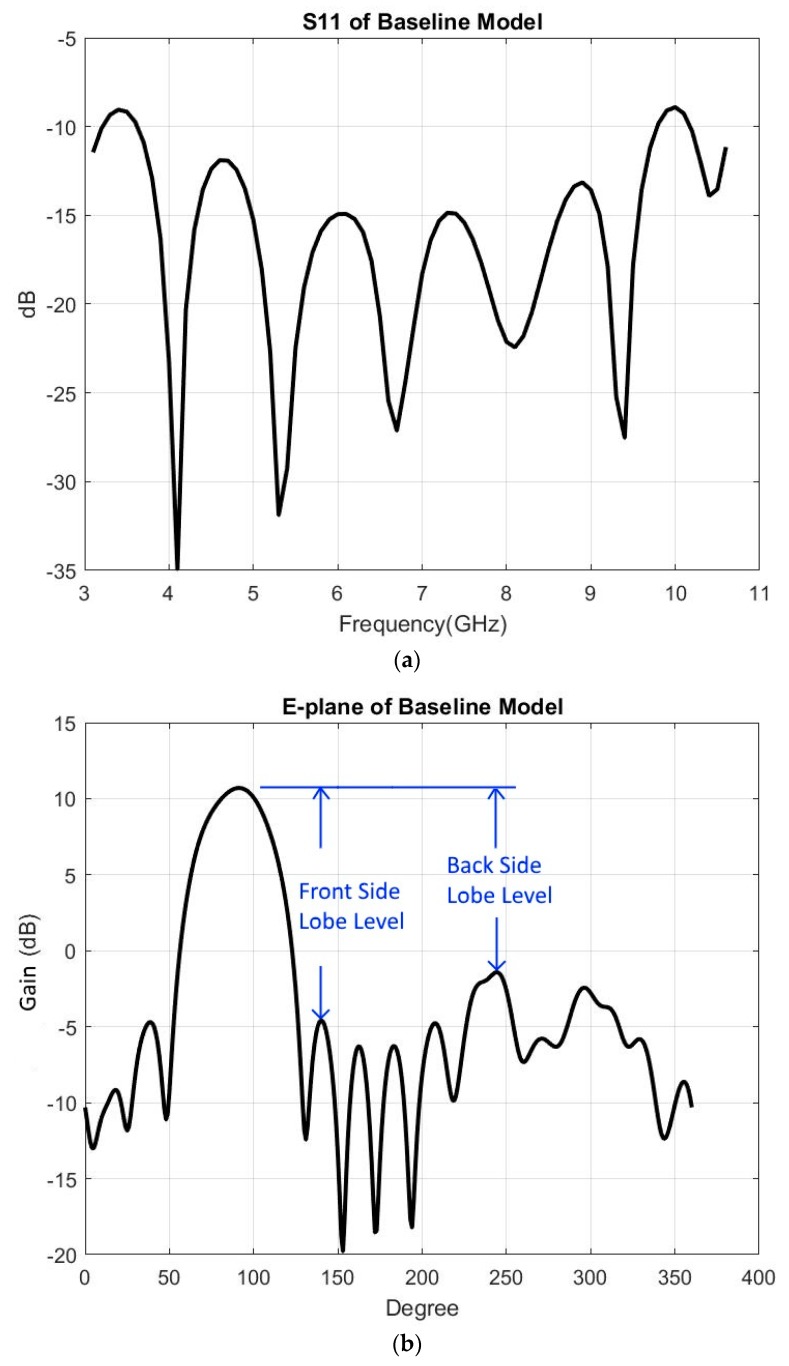

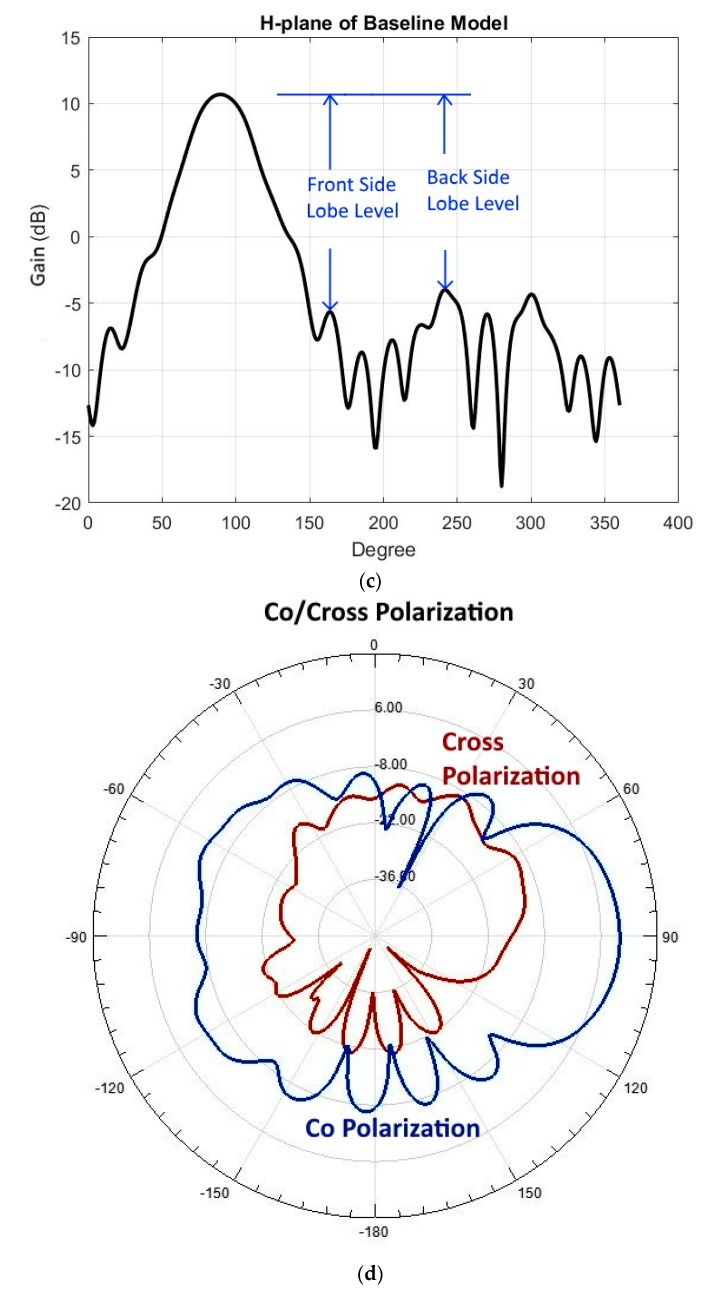
Baseline model characteristics: (**a**) S11, (**b**) E-plane, (**c**) H-plane, and (**d**) co/cross polarization (Blue: copolarization, Red: cross polarization).

Figure 2 shows the baseline model characteristics of S11, the far field radiation pattern on the E-plane and H-plane, the copolarization, and the cross polarization.

### 2.2. Software Tools

The Ansoft HFSS software tool was used in this study. The HFSS software is commonly used by researchers, as it shows good consistency between simulations and actual measurements [9,10,15,22].

### 2.3. Assessment Criteria

Among antenna characteristics, three of them are the most important: return loss (S11), the far field radiation pattern on the E-plane, and the far field radiation pattern on the H-plane. Here, these three characteristics were the assessment objects. Below is a description of the desirable output of these three key antenna characteristics:

1. S11 indicates the power loss caused by the antenna, with less loss being better; −10 dB is a desirable value because it means only 10% of power is lost [8,9,10]. In this study, −9.25 dB (11.89% power loss) was aimed for to leave room for multiple parameter adjustments. Therefore, the potential S11 improvement of the baseline model was 0.75 dB.
2. Far field radiation pattern on the E-plane (“E-plane”) is composed of the main lobe and multiple side lobes. Four important measurements were conducted on the pattern in this study: the measurement of HPBW, gain, side lobe level, and cross polarization discrimination (XPD).
  - HPBW is the degree between two half-power points of the main lobe.
  - Gain is the maximum antenna gain.
  - Side lobe level is defined as the difference in decibels between the main beam peak value and the side lobe peak value. The peak can occur at the same side as the main lobe (front) or at the opposite side (back). For this study, the front was of greater interest because it was the direction in which the object faced. The desirable front side lobe level (FSLL) was greater than 11.5 dB in order to achieve 70% beam efficiency [8,11].
  - XPD is defined as a ratio of the copolar component of the specified polarization compared to the orthogonal cross-polar component over the beamwidth angle [23]. The angles that were examined were 70°, 90°, and 110°. The 90° angle was the on-axial (Y-axis) direction, and the 70° and 110° angles were the general position where the HPBW was located.
3. Far field radiation pattern on the H-plane (“H-plane”) is also composed of the main lobe and multiple side lobes. Its HPBW, gain, and FSLL are of the same importance as those in the E-plane, and the preferred FSLL was also desired to be greater than 11.5 dB to achieve 70% beam efficiency.

The values of the parameters affect the antenna characteristics. The impact of varying the 10 parameters on the abovementioned three antenna characteristics was observed, reviewed, and analyzed. In order to identify the distinctive impact of each individual parameter, only one parameter was changed at any given time. 

## 3. Analysis

This section describes how the value of each parameter in the baseline model was derived. Either they were generated from previous studies, as in the case of slot width ($W_{s}$), microstrip width ($W_{m}$), slot stub radius ($R_{s}$), microstrip stub radius ($R_{m}$), slot opening width ($W_{a}$), tapered length ($L_{t}$), and substrate thickness (h), or if they were not suggested in previous studies, an initial value was randomly picked, then incrementally changed till desirable results were attained, as in the case of ground width ($G_{w}$), ground length ($G_{l}$), and substrate material.

Furthermore, this section analyzes the impact of each parameter’s variations on antenna characteristics in terms of desirable level, as described in Section 2.3.

### 3.1. Slot Width ($W_{s}$) and Microstrip Width ($W_{m}$)

Janaswamy and Schaubert [24] indicated that slot characteristic impedance ($Z_{so}$) was determined by $W_{s}$, $\varepsilon_{r}$, h, and $\lambda_{0}$, where $\lambda_{0}$ is the free space wavelength, and calculated with the following equations:

(1) $$\begin{array}{ll} Z_{so}= & 60+3.69\sin[\frac{(\varepsilon_{r}-2.22)\pi }{2.36}]+133.5\ln(10\varepsilon_{r})\sqrt{\frac{W_{s}}{\lambda_{0}}}\\ & +2.81[1-0.11\varepsilon_{r}(4.48+\ln\varepsilon_{r})](\frac{W_{s}}{h})\ln(100\frac{h}{\lambda_{0}})\\ & +131.1(1.028-\ln\varepsilon_{r})\sqrt{\frac{h}{\lambda_{0}}}+12.48(1+0.18\ln\varepsilon_{r})\frac{\frac{W_{s}}{h}}{\sqrt{\varepsilon_{r}-2.06+0.85{\left(\frac{W_{s}}{h}\right)}^{2}}}. \end{array}$$

Equation (1) is valid when $0.0006\leq \frac{h}{\lambda_{0}}\leq 0.006$, $0.015\leq \frac{W_{s}}{\lambda_{0}}\leq 0.075$, and $2.2\leq \varepsilon_{r}\leq 3.8$. With the same limitation on $\frac{h}{\lambda_{0}}$ and $\frac{W_{s}}{\lambda_{0}}$ but with a different $\varepsilon_{r}$ range, $3.8<\varepsilon_{r}\leq 9.8$, then $Z_{so}$ became

(2) $$\begin{array}{ll} Z_{so}= & 73.6+2.15\varepsilon_{r}+(638.9-31.37\varepsilon_{r}){\left(\frac{W_{s}}{\lambda_{0}}\right)}^{0.6}\\ & +(36.23\sqrt{\varepsilon_{r}{}^{2}+41}-225)\frac{\frac{W_{s}}{h}}{\left(\frac{W_{s}}{h}+0.876\varepsilon_{r}-2\right)}\\ & +0.51(\varepsilon_{r}+2.12)\left(\frac{W_{s}}{h}\right)\ln\left(100\frac{h}{\lambda_{0}}\right)-0.753\varepsilon_{r}\frac{\frac{h}{\lambda_{0}}}{\sqrt{\frac{W_{s}}{\lambda_{0}}}}. \end{array}$$

In addition, the slot width is determined by the print circuit board manufacturer’s processing capability. For FR4 material, such as Isola FR406, it is safe to assume most manufacturers can process the slot width as small as 7 mil (0.178 mm) [25].

The microstrip impedance ($Z_{mo}$) is controlled by the width of the microstrip ($W_{m}$), the copper thickness (t), and the substrate thickness (h). For this study, the copper thickness was set to 0.5 oz., as is mostly used in industry practice [25], and h = 30 mil, which was mechanically rigid enough not to require special fixture for installation. The $Z_{mo}$ was calculated according to the IPC-2141A standard [26]:

(3) $$Z_{mo}=\frac{\eta_{0}}{2\sqrt{2\pi }\sqrt{\varepsilon_{r,eff}+1}}\ln\{1+4\frac{h}{W^{\prime }}[4\left(\frac{14\varepsilon_{r,eff}+8}{11\varepsilon_{r,eff}}\right)\frac{h}{W^{\prime }}+\sqrt{16{\left(\frac{14\varepsilon_{r,eff}+8}{11\varepsilon_{r,eff}}\right)}^{2}{\left(\frac{h}{W^{\prime }}\right)}^{2}+\frac{\varepsilon_{r,eff}+1}{2\varepsilon_{r,eff}}\pi^{2}}]\}$$

where $\eta_{0}$ is the wave impedance of free space, 377 Ω. The $W^{\prime }$ is the effective signal line width:

(4) $$W^{\prime }=W_{m}+\frac{t}{\pi }\ln[\frac{4e}{\sqrt{{\left(\frac{t}{h}\right)}^{2}+{\left(\frac{t}{W_{m}\pi +1.1t\pi }\right)}^{2}}}]\left(\frac{\varepsilon_{r,eff}+1}{2\varepsilon_{r,eff}}\right)$$

where t is the copper thickness (0.5 oz. = 0.7 mil) and $\varepsilon_{r,eff}$ is the effective relative permittivity of the substrate:

(5) $$\varepsilon_{r,eff}=\frac{\varepsilon_{r}+1}{2}+\frac{\varepsilon_{r}-1}{2}[\sqrt{\frac{W_{m}}{W_{m}+12h}}+0.04{\left(1-\frac{W_{m}}{h}\right)}^{2}],\;when\;\frac{W_{m}}{h}<1.$$

**Figure 3:**
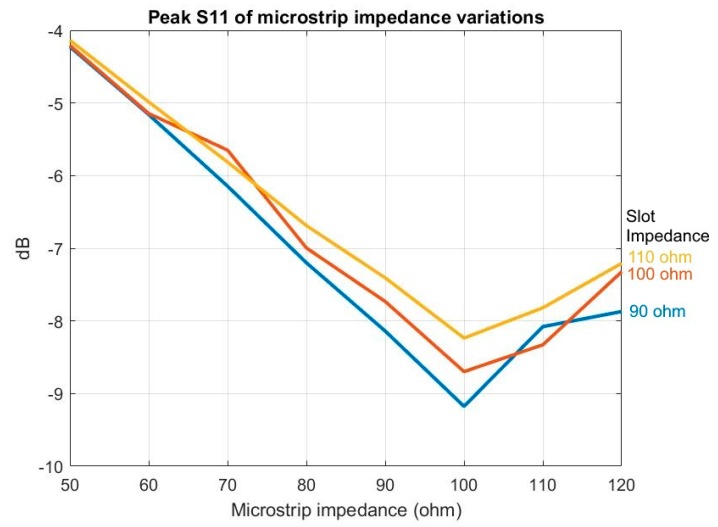
Peak S11 of variances of microstrip impedance (blue: 90-Ω slot impedance, red: 100-Ω slot impedance, and yellow: 110-Ω slot impedance).

TSA utilizes a microstrip-to-slot transition structure to convert the electrical signal (microstrip) to an electromagnetic wave signal (slot) [27]. This transition requires matching $Z_{mo}$ and $Z_{so}$. With the width limited by the manufacturing process to 7 mil, calculation via Equations (2) and (3) returns the minimum $Z_{so}$ as 90 Ω and the maximum $Z_{mo}$ as 120 Ω. To determine the best match, three $Z_{so}$ values—90 Ω ($W_{s}$ = 0.178 mm), 100 Ω ($W_{s}$ = 0.294 mm), and 110 Ω ($W_{s}$ = 0.426 mm)—were evaluated. Multiple $Z_{mo}$ were selected and examined for each $Z_{so}$. Figure 3 shows peak S11 values of different $Z_{so}$.

As indicated in Figure 3, it was discovered that:

1. Varying $Z_{so}$ and $Z_{mo}$ only, the improvement to S11 could not meet the assessment S11 criterion,
2. The lowest S11 peak always occurred at $Z_{mo}$ = 100 Ω, and
3. Lowering $Z_{so}$ would lower the S11 peak value.

Simulation results from the combination of $Z_{so}$ and $Z_{mo}$ indicated that they had little impact on the E-plane and H-plane, nor on HPBW, gain, FSLL, or XPD.

### 3.2. Slot Stub Radius ($R_{s}$) and Microstrip Stub Radius ($R_{m}$)

Slot stub radius $R_{s}$ and microstrip stub radius $R_{m}$ function as the termination points of the microstrip-to-slot transition. It has often been recommended that the length of both stubs should be equal to one quarter of $\lambda_{s}$ [17,28]. However, other researchers [18,29] have suggested that the slot stub length should be $\frac{1}{6}\lambda_{s}$. To explore the possible best length, simulations were performed with $R_{s}$ varied from $\frac{1}{6}\lambda_{s}$ to $\frac{1}{4}\lambda_{s}$ with an increment of $\frac{0.1}{12}\lambda_{s}$ For each $R_{s}$, $R_{m}$ was varied from $\frac{1}{6}\lambda_{s}$ to $\frac{1}{4}\lambda_{s}$ with a $\frac{0.1}{12}\lambda_{s}$ increment. The peak values of S11 for each combination of $R_{s}$ and $R_{m}$ are shown in Table 2.

Table 2 shows that the S11 ≤ −10 dB requirement can be met by adjusting $R_{s}$ and $R_{m}$ alone, but it can only occur in a few combinations (dark green cells in Table 2).

However, to satisfy the S11 assessment criterion of S11 ≤ −9.25 dB as set in this study, $R_{s}$ needs to be $\leq \frac{2.6}{12}\lambda_{s}$ and $R_{m}\leq \frac{2.7}{12}\lambda_{s}$ (light green cells in Table 2).

Variations of $R_{s}$ and $R_{m}$ did not change the HPBW of the E-plane or H-plane, and gain, FSLL, and XPD all stayed the same.

### 3.3. Slot Opening Width ($W_{a}$)

A slot antenna behaves like a standard traveling-wave antenna in that the opening controls the bandwidth. Balanis [17] suggested that the minimum slot opening width ($W_{a,\min}$) should be equal to the wavelength of the highest operating frequency, $W_{a,\min}=\frac{C}{f_{\max}\sqrt{\varepsilon_{r}}}$, where *C* is the speed of light and $\varepsilon_{r}$ is the relative permittivity of the printed circuit board (PCB) material (equal to 4.1 at 5 MHz), and the maximum slot opening width, $W_{a,\max}$, should be between the wavelength of the center operating frequency, $W_{a,\max1}=\frac{C}{f_{0}\sqrt{\varepsilon_{r}}}$, and half of the lowest operating frequency, $W_{a,\max2}=\frac{C}{2f_{\min}\sqrt{\varepsilon_{r}}}$. In this study, $f_{\max}$ = 10.6 GHz, $f_{0}$ = 6.85 GHz, and $f_{\min}$ = 3.1 GHz; thus, $W_{a,\min}$ = 13.97 mm, $W_{a,\max1}$ = 21.61 mm, and $W_{a,\max2}$ = 23.88 mm.

**Figure 4:**
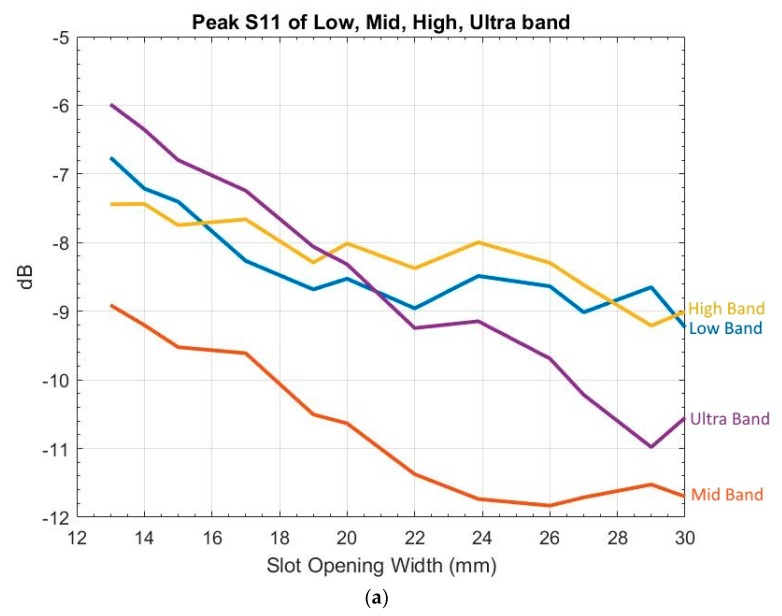

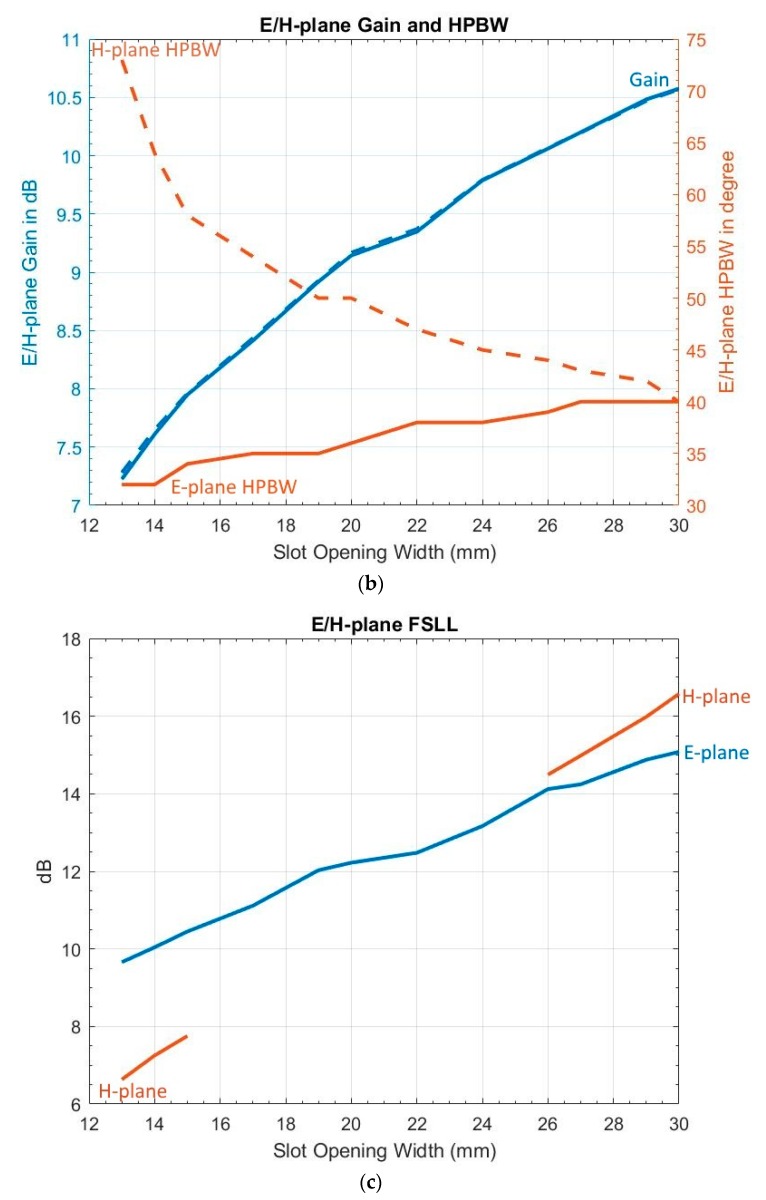
Variation of slot opening width: (**a**) peak S11 of low, mid, high, and ultra bands (blue: low, red: mid, yellow: high, purple: ultra); (**b**) E/H-plane gain, HPBW (in degree) (blue solid: E-plane gain, blue dash: H-plane gain, red solid: E-plane HPBW, red dash: H-plane HPBW); and (**c**) E-plane and H-plane FSLL (blue: E-plane, red: H-plane). Note: The discontinuity on the H-plane FSLL resulted from no detectable front side lobe peak.

To gauge its impact, $W_{a}$ was varied from 13 to 30 mm. When examining S11 results, the UWB frequency range was divided into four groups: 3.1–4.1 GHz (low), 4.1–9.2 GHz (mid), 9.2–10.2 GHz (high), and 10.2–10.6 GHz (ultra), because overshoot over −10 dB occurred mostly in low, high, and ultra bands, while the mid band was most likely to see S11 below −10 dB. The S11 peak of each band versus $W_{a}$ is shown in Figure 4a, the E-plane and H-plane gain and HPBW versus $W_{a}$ in Figure 4b, and the E-plane and H-plane FSLL versus $W_{a}$ in Figure 4c.

Figure 4c shows that, in order to meet assessment criteria b and c, $W_{a,\min}$ has to be greater than 23 mm, which is greater than the 13.97 mm specified by Balanis. In addition, Figure 4 shows continuing improvement of S11, gain, and FSLL beyond $W_{a,\max1}$ and $W_{a,\max2}$.

The continuing improvement occurred beyond the maximum boundary set by Balanis [17]. Also, the fail observed at the minimum boundary could be attributed to the fact that Balanis used a fixed relative permittivity. However, according to Janaswamy and Schaubert [24], relative permittivity should be frequency dependent, not fixed. To discover the real boundary, the frequency-dependent relative permittivity $\varepsilon_{eff}$ should be calculated, which can be done through the following equations provided by Janaswamy and Schaubert [24]:

(6) $$\varepsilon_{eff}=\frac{1}{{\left(\lambda_{s}/\lambda_{0}\right)}^{2}}$$

where $\lambda_{s}$ is the wavelength when passing through PCB material, and $\lambda_{0}$ is the free space wavelength. Therefore, the formula below is valid when $0.0006\leq \frac{h}{\lambda_{0}}\leq 0.006$, $0.015\leq \frac{W_{s}}{\lambda_{0}}\leq 0.075$, and $2.2\leq \varepsilon_{r}\leq 3.8$:

(7) $$\frac{\lambda_{s}}{\lambda_{0}}=1.045-0.365\ln\varepsilon_{r}+\frac{6.3\left(\frac{W_{s}}{h}\right)\varepsilon_{r}{}^{0.945}}{\left(238.64+100\frac{W_{s}}{h}\right)}-[0.148-\frac{8.81(\varepsilon_{r}+0.95)}{100\varepsilon_{r}}]\ln\left(\frac{h}{\lambda_{0}}\right).$$

With the same limitation on $\frac{h}{\lambda_{0}}$ and $\frac{W_{s}}{\lambda_{0}}$ but a different $\varepsilon_{r}$ range, $3.8<\varepsilon_{r}\leq 9.8$, then

(8) $$\begin{array}{ll} \frac{\lambda_{s}}{\lambda_{0}}= & 0.9217-0.277\ln\varepsilon_{r}+0.0322\left(\frac{W_{s}}{h}\right)\sqrt{\frac{\varepsilon_{r}}{\left(\frac{W_{s}}{h}+0.435\right)}}\\ & -0.01\ln\left(\frac{h}{\lambda_{0}}\right)[4.6-\frac{3.65}{\varepsilon_{r}{}^{2}\sqrt{\frac{W_{s}}{\lambda_{0}}}\left(9.06-100\frac{W_{s}}{\lambda_{0}}\right)}]. \end{array}$$

**Figure 5:**
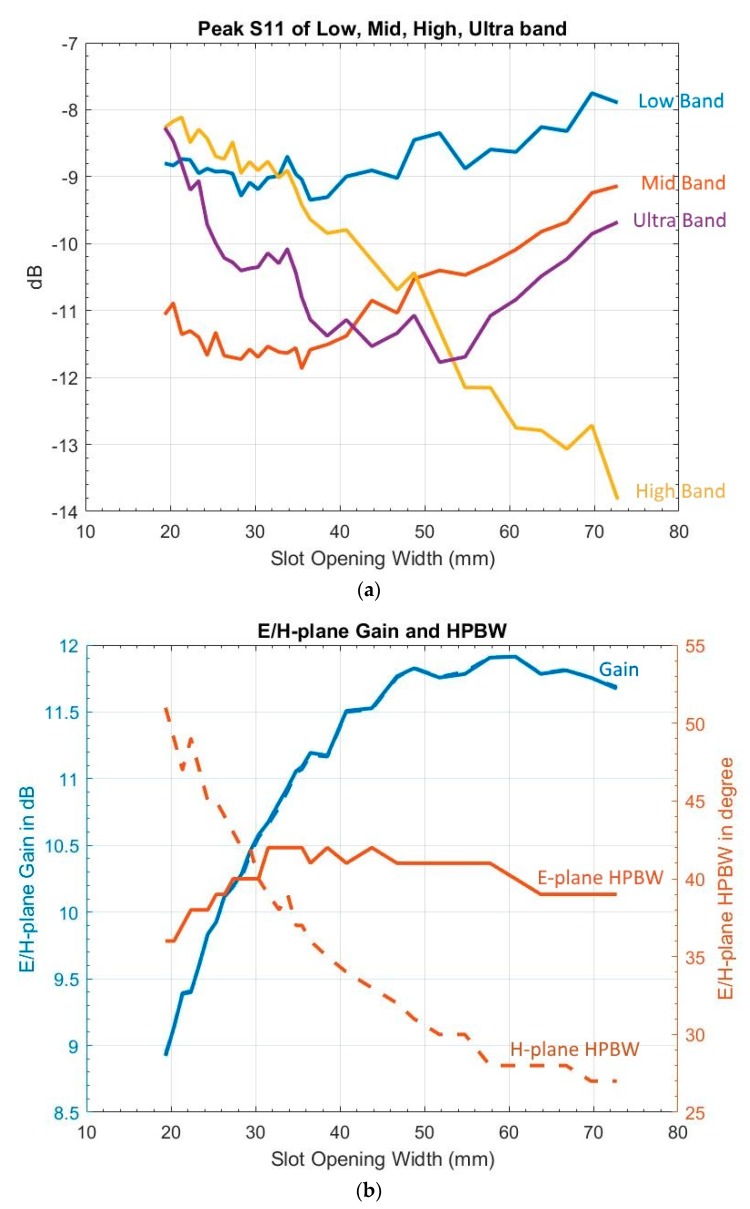

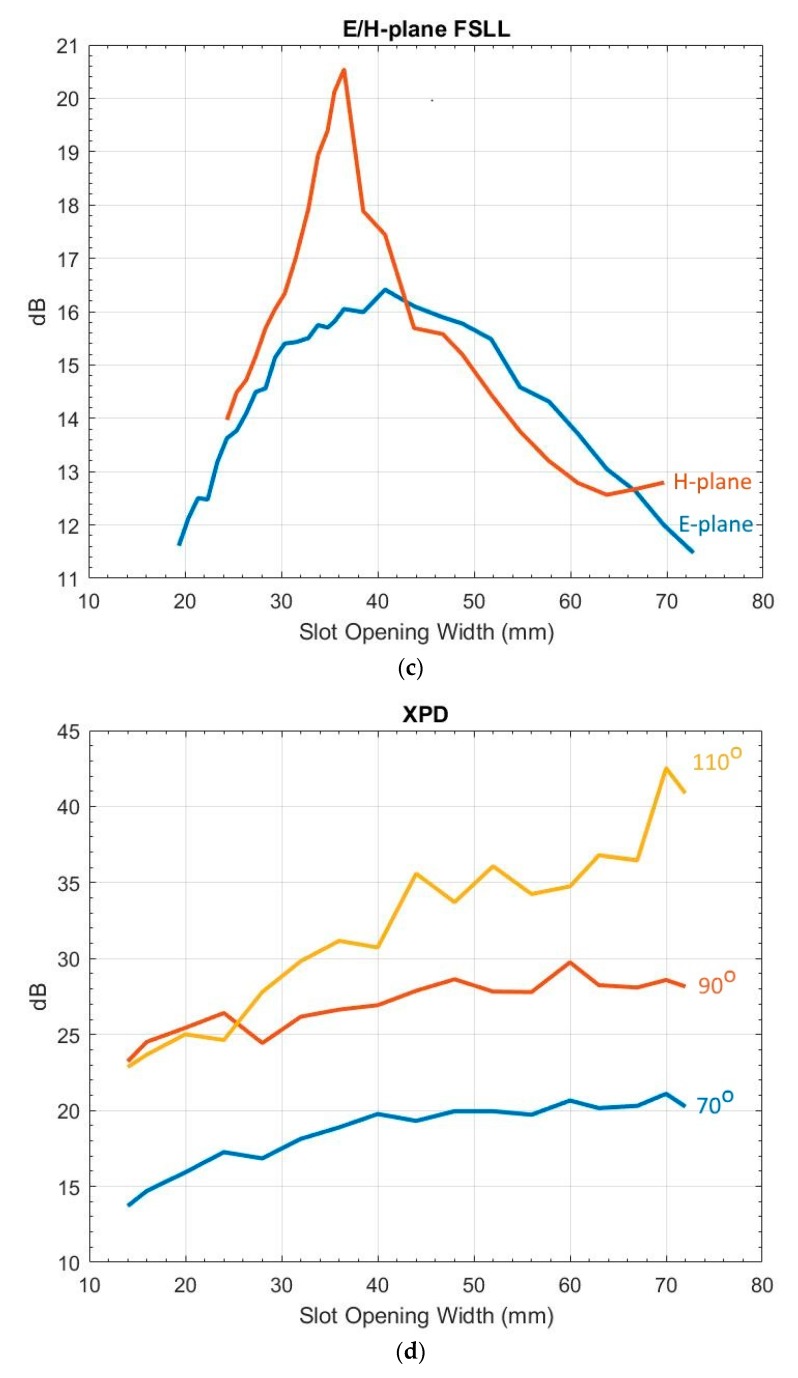
Variation of slot opening width based on $\varepsilon_{eff}$: (**a**) peak S11 of low, mid, high, and ultra bands (blue: low, red: mid, yellow: high, purple: ultra); (**b**) E/H-plane gain, HPBW (blue solid: E-plane gain, blue dash: H-plane gain, red solid: E-plane HPBW, red dash: H-plane HPBW); (**c**) E/H plane FSLL, (blue: E-plane, red: H-plane); and (**d**) XPD (blue: 70°, red: 90°, yellow: 110°). Note: Missing data on H-plane FSLL resulted from no detectable front side lobe peak.

To discover the new $W_{a}$ setting based on the calculated $\varepsilon_{eff}$, simulations were performed with $W_{a}$ varying from 19 to 73 mm. S11 peaks of each band are shown in Figure 5a, gain and HPBW of the E- and H-plane in Figure 5b, E/H-plane FSLL in Figure 5c, and XPD at 70°, 90°, and 110° in Figure 5d. Figure 5 reveals that

1. Changing $W_{a}$ alone will not satisfy the S11 assessment criterion,
2. Increasing $W_{a}$ will decrease the peak S11 values within ultra band range,
3. Increasing $W_{a}$ will increase antenna gain,
4. $W_{a}$ has to be between 19 and 70 mm to satisfy the E-plane and H-plane FSLL assessment criteria. Therefore, it is safe to assume
  (9) $$W_{a,\min}=\frac{C}{f_{\max}\sqrt{\varepsilon_{eff}}}$$
  because using $\varepsilon_{eff}$ instead of $\varepsilon_{r}$ resulted in $W_{a,\min}$ = 19.91 mm, which coincided with the simulation results. Meanwhile, $W_{a,\max}$ was changed to
  (10) $$W_{a,\max1}=2\frac{C}{f_{0}\sqrt{\varepsilon_{eff}}}$$
  and
  (11) $$W_{a,\max2}=\frac{C}{f_{\min}\sqrt{\varepsilon_{eff}}}$$
  which corresponded to 63.01 and 69.62 mm (~70 mm), respectively, and
5. Changing $W_{a}$ has some but no significant impact on XPD.

### 3.4. Tapered Length ($L_{t}$)

**Figure 6:**
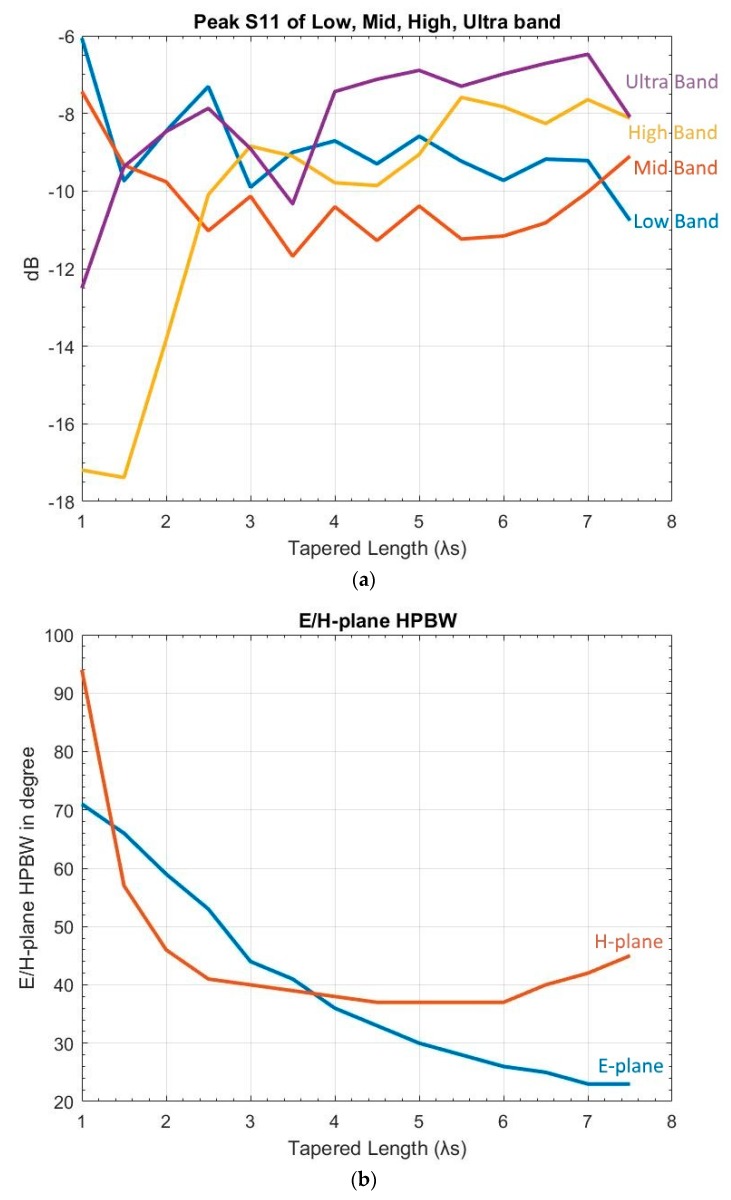

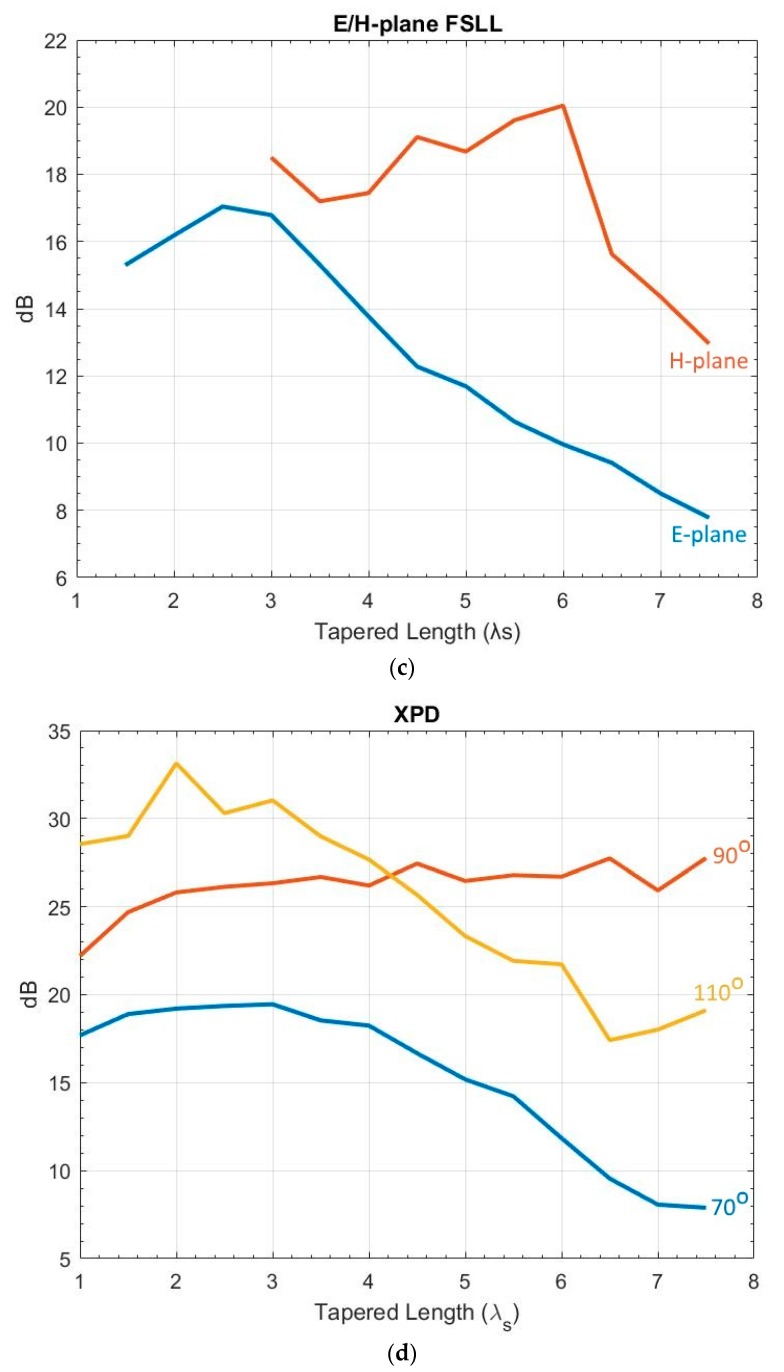
Variation of tapered length: (**a**) peak S11 of low, mid, high, and ultra bands (blue: low, red: mid, yellow: high, purple: ultra); (**b**) E/H-plane HPBW (blue: E-plane, red: H-plane); (**c**) E/H plane FSLL (blue: E-plane, red: H-plane); and (**d**) XPD (blue: 70°, red: 90°, yellow: 110°). Note: Missing data on H-plane FSLL resulted from no detectable front side lobe peak.

The tapered length is known to affect E-plane HPBW—the longer the length, the smaller the E-plane HPBW. A previous study [11] reported that the benefit of a longer length diminished when $L_{t}\geq 6\lambda_{s}$. However, that study focused on a much higher frequency range (26.5 to 40 GHz) than in this study. To confirm whether the same limitation also applied to the UWB range, simulations were performed with $L_{t}$ ranging from $1.0\lambda_{s}$ to $7.5\lambda_{s}$, at an increment of $0.5\lambda_{s}$. The results showed that $L_{t}$ variation did not have a definite impact on S11 nor on the H-plane. However, to meet the E-plane side lobe assessment criterion, $L_{t}$ had to be $5\lambda_{s}\geq L_{t}\geq 1.5\lambda_{s}$. On the other hand, increasing $L_{t}$ would decrease XPD at 70° and 110° but not at 90°. Figure 6 shows the results.

### 3.5. Ground Width ($G_{w}$) and Ground Length ($G_{l}$)

It is desirable for most antenna designs to have infinite ground, but it is not possible in reality. To find the appropriate values of ground width, simulations were performed on various sizes, starting from $\frac{1}{4}\lambda_{s}$ to $2\lambda_{s}$, with an increment of $\frac{1}{8}\lambda_{s}$. 

**Figure 7:**
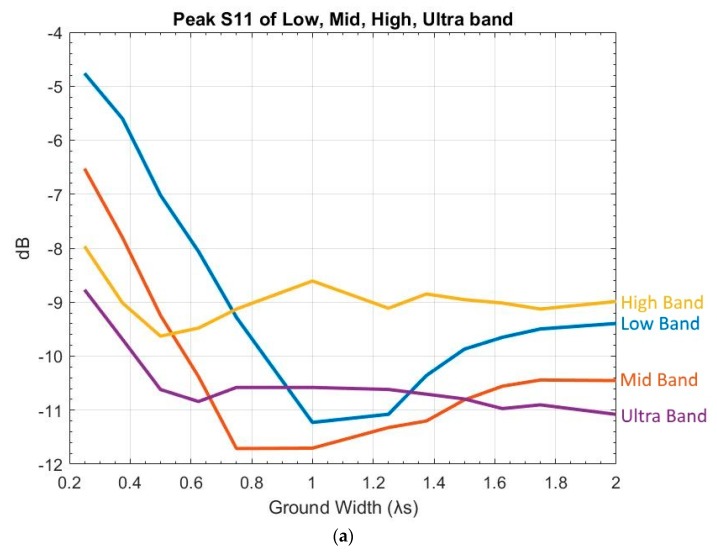

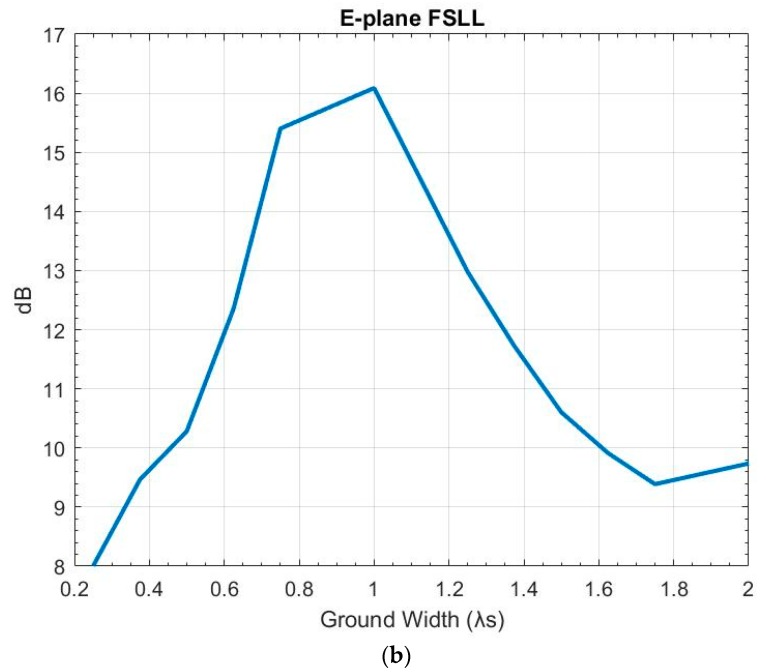
Variation of ground width: (**a**) peak S11 of low, mid, high, and ultra bands (blue: low, red: mid, yellow: high, purple: ultra); and (**b**) E-plane FSLL.

Results of $G_{w}$ variation are shown in Figure 7, and they indicate that, to meet S11 and FSLL criteria, $G_{w}$ has to be between $\frac{1}{2}\lambda_{s}$ and $1\frac{1}{2}\lambda_{s}$. In addition,

1. Increasing $G_{w}$ will decrease overshoot in both low and ultra bands, with higher reduction in low bands, and
2. $G_{w}$ variation has little impact on H-plane, E-plane HPBW, gain, or XPD.

**Figure 8:**
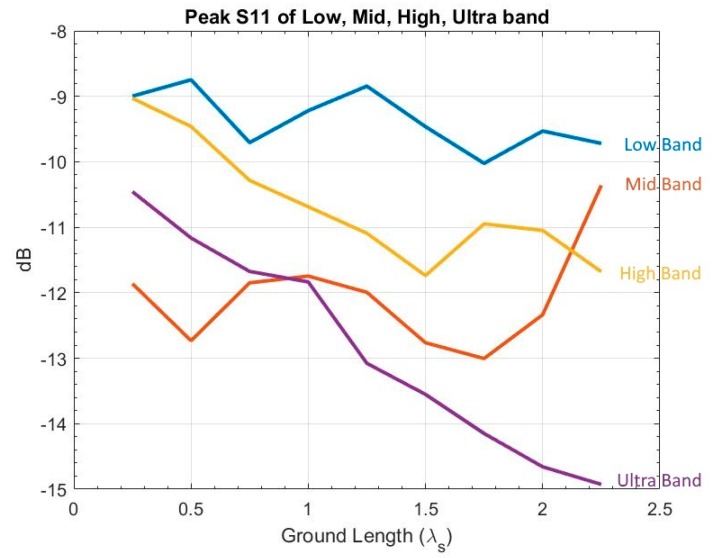
Variation of ground length (blue: low band, red: mid band, yellow: high band, purple: ultra band).

A similar setting to $G_{w}$ was chosen for $G_{l}$ simulation, starting from $\frac{1}{4}\lambda_{s}$ to $2\frac{1}{4}\lambda_{s}$, with a $\frac{1}{8}\lambda_{s}$ increment. Results (Figure 8) indicate that:

1. Increasing $G_{l}$ will decrease the S11 peak in high and ultra bands,
2. When $\frac{3}{8}\lambda_{s}\leq G_{l}\leq \lambda_{s}$ or $G_{l}\geq 1\frac{3}{4}\lambda_{s}$, it will meet the S11 criterion, and
3. Variation of $G_{l}$ has little impact on E-plane, H-plane, or XPD (not shown).

### 3.6. Substrate Thickness (h)

Substrate thickness affects antenna gain and side lope level [8]. However, there is no consensus on what the thickness range should be, as there is no consensus on the effective dielectric thickness, $h_{eff}$, normalized over $\lambda_{0}$. The $h_{eff}$ is related to h by

(12) $$h_{eff}=\left(\sqrt{\varepsilon_{r}}-1\right)\frac{h}{\lambda_{0}}.$$

One study proposed that it should be from 0.005 to 0.03 [11], while another proposed 0.005 to 0.01 [8]. 

**Figure 9:**
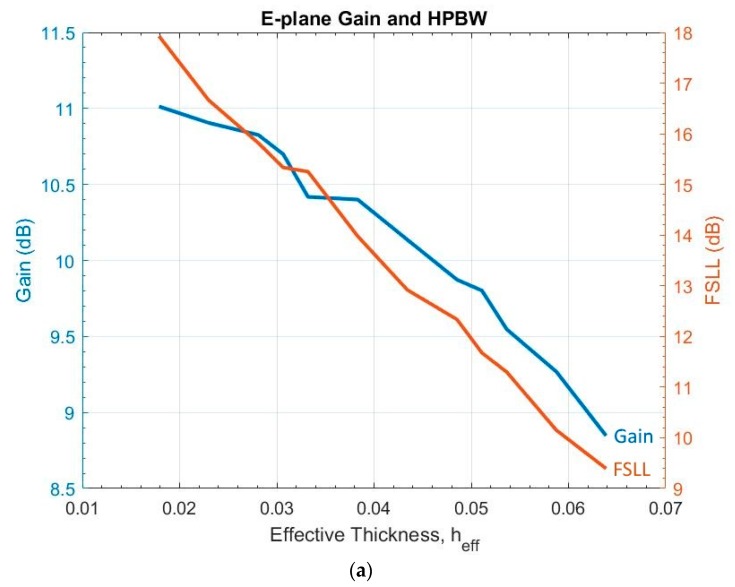

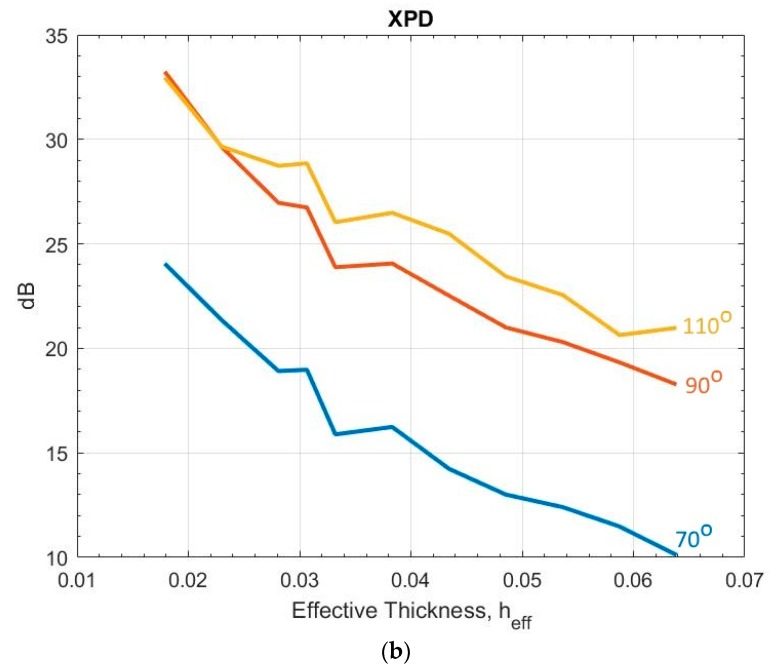
Variations of substrate effective thickness: (**a**) E-plane gain and HPBW; and (**b**) XPD (blue: 70°, red: 90°, yellow: 110°).

Since previous studies [8,11] calculated $h_{eff}$, instead of h, for the purpose of comparison, this study also gauged the impact on the antenna by the expression of $h_{eff}$ instead of h. Simulations on thickness were performed with 5-mil increments, starting at 17.5 mil ($h_{eff}$ = 0.01788) and ending at 62.5 mil ($h_{eff}$ = 0.06387). Microstrip trace width is affected by substrate thickness according to Equation (3). To maintain 7 mil of microstrip width (minimum manufacturing process allowance), the smallest substrate thickness was 17.5 mil. Figure 9 shows the $h_{eff}$ impact on E-plane gain, side lobe level, and XPD. It reveals that:

1. Increasing the thickness causes a slight gain variation (1.5 dB),
2. FSLL will fall below −11.5 dB when $h_{eff}\geq 0.052$,
3. $h_{eff}$ impact on S11 is less prominent but generally follows the same trend as the E-plane,
4. $h_{eff}$ has little impact on the H-plane, and
5. Increasing $h_{eff}$ reduces XPD on all three angles.

### 3.7. Substrate Material

Not only do different materials have different relative permittivity ($\varepsilon_{r}$) and tangent loss (*tanδ*), the difference exists even among different types of the same material. Consequently, the question arises as to whether antennas of the same size and shape will behave the same when utilizing different materials or types. In this study, two materials—FR4 (FR406) [21] and FR4-like (FR408) [30], each with two types (106 and 7628)—were examined. In most cases, type 106 possessed the lowest $\varepsilon_{r}$ and type 7628 the highest within the same family. The different relative permittivity and tangent loss of the examined materials are shown in Table 3.

**Figure 10:**
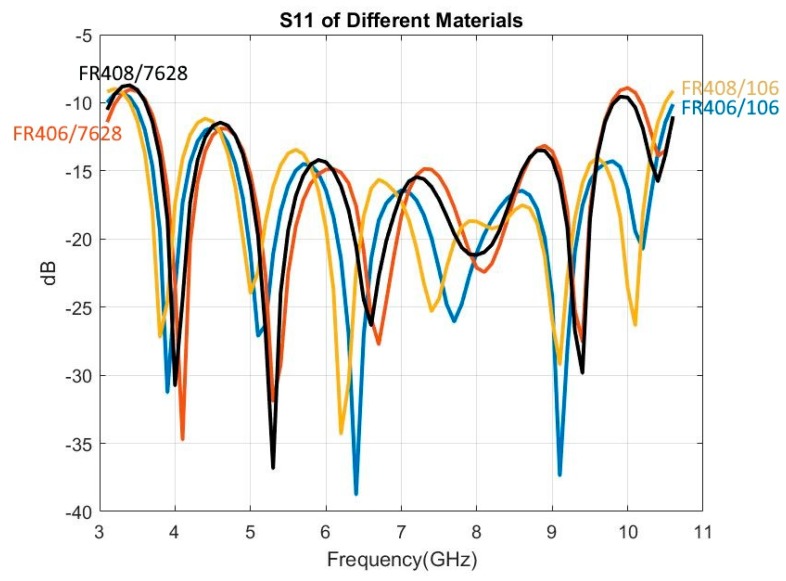
Materials impact on S11 (blue: FR406 type 106, red: FR406 type 7628, yellow: FR408 type 106, black: FR408 type 7628).

The simulation results (Figure 10) from using different materials show that materials or their types do not make any noticeable difference as long as slot and microstrip impedances and substrate thicknesses stay the same. All four material–type combinations have similar S11, E/H-plane gain, HPBW, and XPD. (Note: only S11 is shown in Figure 10).

## 4. Results and Discussions

Compared with previous studies, this study conducted a more comprehensive examination of the 10 parameters, leading to a refined range of values for each parameter in LTSA design. In summary, the following guidelines are recommended when designing a UWB LTSA:

1. Lowest S11 peak always occurs at $Z_{mo}$ = 100 Ω,
2. Lowering $Z_{so}$ would lower S11 peak value,
3. Slot stub radius ($R_{s}$) should be less than $\frac{2.6}{12}\lambda_{s}$,
4. Microstrip stud radius ($R_{m}$) should be less than $\frac{2.7}{12}\lambda_{s}$,
5. Slot opening width ($W_{a}$) should be between $\max(W_{a,\max1},W_{a,\max2})$ and $W_{a,\min}$,
6. Tapered Length ($L_{t}$) should be between $1.5\lambda_{s}$ and $5\lambda_{s}$,
7. Ground width ($G_{w}$) should be between $\frac{1}{2}\lambda_{s}$ and $1\frac{1}{2}\lambda_{s}$,
8. Ground Length ($G_{l}$) has to be between $\frac{3}{8}\lambda_{s}$ and $\lambda_{s}$, or greater or equal to $1\frac{3}{4}\lambda_{s}$,
9. The effective substrate thickness ($h_{eff}$) should be <0.052, and
10. Substrate materials have little impact on LTSA performance as long as the same thickness is maintained.

Table 4 lists the differences in recommended settings between this study and the previous studies that have been reviewed:

## 5. Conclusions

This study examined and analyzed all 10 primary parameters regarding their individual impact on LTSA design. The result is a set of recommendations for LTSA design to operate within the UWB frequency range. Unlike previous studies, which only focused on a certain set of these 10 parameters, this paper provides a comprehensive recommendation for all 10 parameters, as laid out in the Results and Discussions section, which, if followed, should result in a functional LTSA antenna design. This set of recommendation can also serve as a base for further optimization, if desired. When used for optimization, the results from this study can guide the direction of changes when multiple parameters need to be adjusted simultaneously. Furthermore, this set of recommendations can be applied to other tapered slot antenna designs, as they all share, although are not defined completely, by these 10 parameters. 

## Figures and Tables

**Table 1:** Baseline model parameters.

Parameter	Setting	Notes
Slot width ($W_{s}$)	0.178 mm	= minimum PCB manufacturer’s processing capability
Microstrip width ($W_{m}$)	0.354 mm	= width that generated 100 Ω impedance
Slot stud radius ($R_{s}$)	6.0386 mm	= $\frac{2.3}{12}\lambda_{s}$, where $\lambda_{s}$ is the wavelength of the center frequency when passing through the PCB material
Slot stud opening angle	90 degrees	Fixed, not varied
Microstrip stud radius ($R_{m}$)	7.0888 mm	= $\frac{2.7}{12}\lambda_{s}$
Microstrip stud opening angle	90 degrees	Fixed, not varied
Slot opening width ($W_{a}$)	31.5056 mm	= $\lambda_{s}$
Tapered length ($L_{t}$)	110.2695 mm	= $3.5\lambda_{s}$
Ground width ($G_{w}$)	23.6292 mm	= $\frac{3}{4}\lambda_{s}$
Ground length ($G_{l}$)	7.8764 mm	= $\frac{1}{4}\lambda_{s}$
Substrate thickness (h)	30 mil	
Substrate material	FR406 Type 7628	FR4 PCB material manufactured by Isola Corporation

Additional note on substrate material: FR4 was selected for this study because, in addition to being the most common material for PCB [19], its characteristics are stable within the UWB frequency range [20]. The relative permittivity ($\varepsilon_{r}$) and loss tangent (tan*δ*) of material used in this study were based on the manufacturer’s datasheet [21], and their values were frequency dependent.

**Table 2:** Peak S11 values (in dB) of $R_{s}$, $R_{m}$ combinations.

	$R_{s}$	$\frac{1}{6}\lambda_{s}$	$\frac{2.1}{12}\lambda_{s}$	$\frac{2.2}{12}\lambda_{s}$	$\frac{2.3}{12}\lambda_{s}$	$\frac{2.4}{12}\lambda_{s}$	$\frac{2.5}{12}\lambda_{s}$	$\frac{2.6}{12}\lambda_{s}$	$\frac{2.7}{12}\lambda_{s}$	$\frac{2.8}{12}\lambda_{s}$	$\frac{2.9}{12}\lambda_{s}$	$\frac{1}{4}\lambda_{s}$
$R_{m}$												
$\frac{1}{6}\lambda_{s}$		−10.34	−9.38	−9.80	−9.20	−8.95	−8.90	−8.66	−8.32	−8.30	−8.19	−8.08
$\frac{2.1}{12}\lambda_{s}$		−10.23	−9.80	−9.56	−9.29	−6.82	−8.76	−8.70	−8.50	−8.06	−8.30	−8.30
$\frac{2.2}{12}\lambda_{s}$		−10.00	−9.20	−9.23	−9.27	−9.22	−8.87	−9.76	−7.96	−8.43	−8.35	−8.32
$\frac{2.3}{12}\lambda_{s}$		−9.77	−8.08	−9.33	−9.18	−9.17	−8.84	−10.21	−8.71	−8.00	−7.59	−7.90
$\frac{2.4}{12}\lambda_{s}$		−9.59	−9.40	−9.49	−9.15	−8.96	−8.66	−8.61	−8.60	−8.48	−8.32	−8.21
$\frac{2.5}{12}\lambda_{s}$		−9.33	−9.44	−9.35	−9.15	−9.10	−8.80	−8.20	−8.66	−8.66	−8.48	−8.44
$\frac{2.6}{12}\lambda_{s}$		−9.18	−9.20	−9.30	−9.08	−8.96	−7.72	−8.72	−8.70	−8.51	−8.51	−7.90
$\frac{2.7}{12}\lambda_{s}$		−7.43	−9.15	−9.26	−8.70	−9.08	−8.74	−8.77	−8.67	−7.68	−8.59	−6.90
$\frac{2.8}{12}\lambda_{s}$		−7.74	−7.98	−7.90	−8.10	−8.02	−8.36	−8.57	−7.95	−8.63	−8.34	−8.01
$\frac{2.9}{12}\lambda_{s}$		−6.24	−5.36	−6.54	−7.20	−7.41	−7.53	−8.00	−8.39	−8.74	−8.11	−8.00
$\frac{1}{4}\lambda_{s}$		−4.48	−4.42	−5.14	−5.51	−5.90	−6.31	−5.64	−6.82	−6.72	−6.05	−6.24

**Table 3:** Relative permittivity and tangent loss.

Material	$\varepsilon_{r}\;\mathrm{at}\;5\;\mathrm{MHz}$	*tanδ* at 5 MHz
FR406 Type 106	3.39	0.023
FR406 Type 7628	4.1	0.016
FR408 Type 106	3.23	0.015
FR408 Type 7628	3.94	0.011

**Table 4:** Comparison with previous studies.

Para-Meter	Previous Studies		This Study
	Range Recommended	Examined Against Assessment Criteria of This Study	Range Recommended
$W_{s}$	N/A	N/A	$Z_{so}<100\;\Omega$
$W_{m}$	N/A	N/A	$Z_{mo}=100\;\Omega$
$R_{s},R_{m}$	$\frac{1}{6}\lambda_{s}$ [18] or $\frac{1}{4}\lambda_{s}$ [28]	S11 passed at $\frac{1}{6}\lambda_{s}$ but failed at $\frac{1}{4}\lambda_{s}$	$R_{s}\leq \frac{2.6}{12}\lambda_{s}$ $R_{m}\leq \frac{2.7}{12}\lambda_{s}$
$W_{a}$	Between $\frac{C}{f_{\max}\sqrt{\varepsilon_{r}}}$ and $\max(\frac{C}{2f_{\min}\sqrt{\varepsilon_{r}}},\frac{C}{f_{0}\sqrt{\varepsilon_{r}}})$ [17]	Failed at $\frac{C}{f_{\max}\sqrt{\varepsilon_{r}}}$ Passed beyond $\max(\frac{C}{2f_{\min}\sqrt{\varepsilon_{r}}},\frac{C}{f_{0}\sqrt{\varepsilon_{r}}})$	between $\frac{C}{f_{\max}\sqrt{\varepsilon_{eff}}}$ and $\max(\frac{C}{f_{\min}\sqrt{\varepsilon_{eff}}},\frac{2C}{f_{0}\sqrt{\varepsilon_{eff}}})$
$L_{t}$	$<6\lambda_{s}$ [11]	$<1.5\lambda_{s}$—S11 failed $>5\lambda_{s}$—FSLL failed	$1.5\lambda_{s}\leq L_{t}\leq 5\lambda_{s}$
$G_{w}$	N/A	N/A	$\frac{1}{2}\lambda_{s}\leq G_{w}\leq 1\frac{1}{2}\lambda_{s}$
$G_{l}$	N/A	N/A	$\frac{3}{8}\lambda_{s}\leq G_{l}\leq \lambda_{s}$ or $G_{l}\geq 1\frac{3}{4}\lambda_{s}$
$h_{eff}$	between 0.005 and 0.03 [11] or between 0.005 and 0.01 [8]	Pass	$h_{eff}<0.052$
*FR4 Material*	N/A	N/A	Negligible impact

A note on materials: Previous studies were performed on substrate materials other than FR4, such as Duriod^®^ 6140 [8,11], alumina [17,28], RT/Duriod^®^ 5870 [17], and RT/Duriod^®^ 6010 [18], which might partially explain the discrepancies in outcomes between this study and the others.
